# Simultaneous Preparation of Abundant Flavonol Triglycosides from Tea Leaves

**DOI:** 10.3390/molecules25215140

**Published:** 2020-11-04

**Authors:** Zhou-Tao Fang, Yi-Qing Lv, Chu-Jun Song, Jing Jin, Jian-Liang Lu, Hai-Rong Xu, Jian-Hui Ye

**Affiliations:** 1 Zhejiang University Tea Research Institute, Hangzhou 310058, China; ztfang@zju.edu.cn (Z.-T.F.); yiqinglv@zju.edu.cn (Y.-Q.L.); 21716161@zju.edu.cn (C.-J.S.); jllu@zju.edu.cn (J.-L.L.); 2 Zhejiang Agricultural Technical Extension Center, 29 Fengqidong Road, Hangzhou 310000, China; zdcxjj@126.com

**Keywords:** *Camellia sinensis*, flavonol glycosides, tri-glycosides, preparative HPLC, isolation, NMR measurement

## Abstract

Flavonol glycosides are important components of tea leaves, contributing to the bioactivities as well as bitterness and astringency of tea. However, the standards of many flavonol triglycosides are still not available, which restricts both sensory and bioactivity studies on flavonol glycosides. In the present study, we established a simultaneous preparation method of seven flavonol triglycoside individuals from tea leaves, which consisted of two steps: polyamide column enrichment and preparative HPLC isolation. The structures of seven flavonol triglycoside isolates were identified by mass and UV absorption spectra, four of which were further characterized by nuclear magnetic resonance spectra, namely, quercetin-3-*O*-glucosyl-rhamnosyl-glucoside, quercetin-3-*O*-rhamnosyl-rhamnosyl-glucoside, kaempferol-3-*O*-glucosyl-rhamnosyl-glucoside and kaempferol-*O*-rhamnosyl-rhamnosyl-glucoside. The purities of all isolated flavonol triglycosides were above 95% based on HPLC, and the production yield of total flavonol glycosides from dry tea was 0.487%. Our study provides a preparation method of flavonol triglycosides from tea leaves, with relatively low cost of time and solvent but high production yield.

## 1. Introduction

Green tea is a widely consumed beverage over the world, which has unique flavor due to the rich presence of secondary metabolites in tea leaves, for example, catechins, flavonol glycosides, caffeine and amino acids [1,2]. Catechin compounds and flavonol glycosides are the major phenolic compounds in tea leaves accounting for 70~80% and ~13% of tea polyphenols, which are associated with many health benefits, such as antioxidant, anti-inflammatory and anti-tumor effects, as well as the bitterness and astringency of tea infusion [3,4,5,6]. Despite the much lower concentration compared with catechins, flavonol glycosides with mellow and astringent taste are considered as essential constituents to the bitterness and astringency of tea infusion, due to its extremely low threshold and enhancing effect on the bitterness of caffeine [7,8]. The threshold of flavonol glycosides ranged from 0.001 μmol/L to 19.80 μmol/L, which were much lower than 190–930 μmol/L of catechins and 13–26 μmol/L of theaflavins [9]. In addition, there is increasing interest in the pharmacological functions of flavonol glycosides on account of their higher stabilities under basic condition compared with catechins and multiple bioactive hydroxyl groups. It was reported that flavonoid glycosides inhibited the activity of α-glucosidase and α-amylase [10], and inhibited the proliferation of human oral squamous carcinoma SCC-9 cells [11]. *O*-glycosylation can enhance certain types of biological benefits such as anti-HIV activity, antirotavirus activity, antistress activity, antiobesity activity, and antiallergic activity [12].

Up to date, more than 20 flavonol glycosides were identified in tea leaves [13]. The majority of flavonol glycosides in tea leaves are the glycosyl derivatives of quercetin, kaempferol and myricetin, with mono-, di- or tri- glycoside moiety at C-3 position. Although the composition of flavonol glycosides in fresh tea leaves was affected by many factors like tea cultivar, plucking standard and growing season, tri-glycoside are the most abundant flavonol glycoside group in tea leaves [14]. However, many flavonol glycosides are still quantified relatively due to the lack of the corresponding commercial standard compounds, especially tri-glycosides, which leads to the difficulties in comparing the results of different studies and achieving the absolute contents of diverse flavonol glycosides. Besides, the limited access to individual flavonol glycosides also restrains the studies on the sensory characteristics and biological functions of flavonol glycosides. Hence, it is important to develop a bulk preparation method of individual flavonol glycosides.

Several attempts have been made to isolate individual flavonol glycosides by using high performance liquid chromatography (HPLC) and preparative HPLC. Scharbert et al. used HPLC to isolate 14 flavonol glycosides in several runs [9]; however, this method is not practical for large-scale production due to its time- and solvent-consuming as well as low production efficiency. Engelhard et al. developed an isolation procedure of tri-glycosides from tea leaves, including polyamide column pre-purification, Sephadex LH-20 column enrichment and preparative HPLC isolation, while only one to two individual flavonol glycosides were achieved on one run [15,16,17]; however, only around 4 mg quercetin-3-*O*-glucosyl-rhamnosyl-galactoside (Q-gal-rha-glu) and 4 mg kaempferol-3-*O*-glucosyl-rhamnosyl-galactoside (K-gal-rha-glu) were obtained from 80 g black tea [16]. Besides, four mono-glycosides and one di-glycoside were isolated from the tea flower through solvent partitioning and two runs preparative HPLC [3]. The repeated use of preparative HPLC or application of HPLC for laboratory-scale isolation was the major reason for the extremely low production efficiency of flavonol glycosides.

In the present study, we developed a two stepwise method for simultaneous preparation of tri-glycosides from tea leaves, comprising flavonol glycoside enrichment by polyamide column and preparative HPLC separation. Ultra-high-performance liquid chromatography–diode array detector–tandem mass spectrometry (UPLC–DAD–MS/MS) were used to identify and quantify flavonol glycosides. There were seven individual flavonol glycosides isolated by preparative HPLC, amongst which four abundant tri-glycosides were further characterized by nuclear magnetic resonance (NMR) measurement.

## 2. Results and Discussion

### 2.1. Optimization of Uploading Volume on the Preparation of Flavonol Glycoside-Enriched Fraction

**Figure 1:**
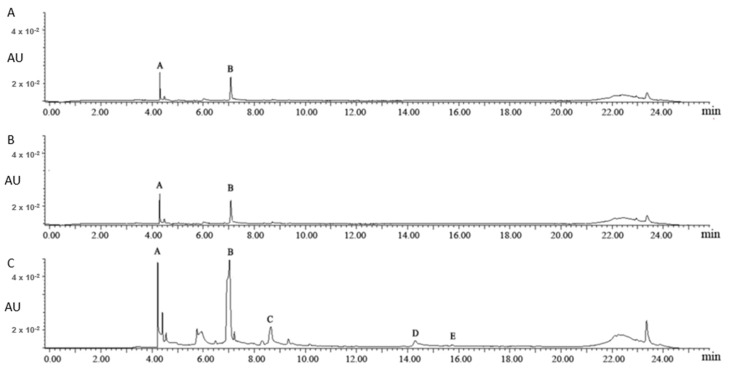
The ultra-high-performance liquid chromatography (UPLC) chromatograms of the aqueous fractions of different uploading volumes: 1 mL (**A**), 2.5 mL (**B**) and 5.0 mL (**C**). Note: Peak A: 3-galloylquinic acid; Peak B: caffeine; Peak C: (−)-epigallocatechin gallate; Peak D: (−)-epicatechin gallate; Peak E: kaempferol-3-*O*-glucosyl-rhamnosyl-glucoside. Peaks B, C, and D were identified by authentic standards; Peaks A and E were identified based on MS and references.

Figure 1 shows the UPLC chromatograms of the aqueous fractions of different uploading volumes. As the uploading volumes were 1 mL and 2.5 mL, 3-galloylquinic acid (Peak A) and caffeine (Peak B) were the major chemical compounds in the aqueous fractions, based on the reported identification information of 3-galloylquinic acid [18]. As the uploading volume further increased to 5 mL, (−)-epigallocatechin gallate (EGCG, Peak C), (−)-epicatechin gallate (ECG, Peak D), and kaempferol-3-*O*-glucosyl-rhamnosyl-glucoside (K-glu-rha-glu, Peak E) were detected in the aqueous fraction, suggesting an overloading of flavonoids on polyamide column. Thus, 2.5 mL was selected for the following studies to achieve a sufficient adsorption and utilization of flavonol glycosides in tea leaves.

### 2.2. Impact of Elution Method on the Composition of Flavonol Glycoside-Enriched Fraction

Table 1 shows the chemical compositions of different fractions isolated by different elution methods. For method A, 60% methanol fraction was the flavonol glycoside-enriched fraction that contained 140.13 ± 1.56 µg/mL of total flavonol glycosides (TFG), while EGC and (−)-epicatechin (EC) were simultaneously eluted, with the concentrations of 240.79 ± 12.19 µg/mL and 35.11 ± 2.39 µg/mL.

**Figure 2:**
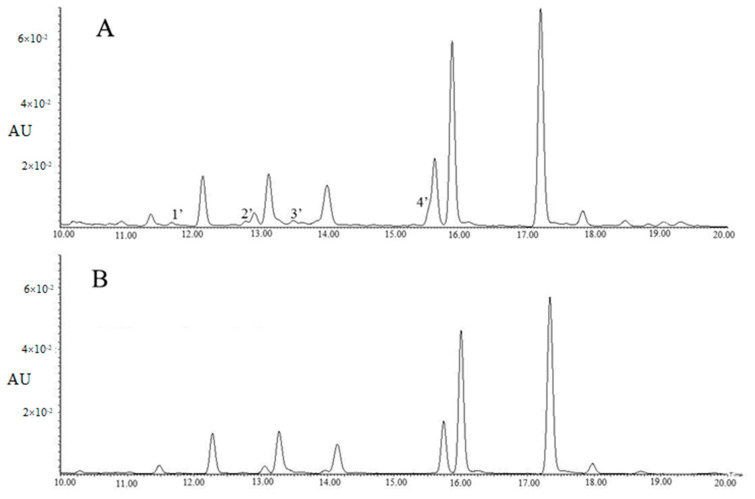
The UPLC chromatograms of 60% methanol fraction (**A**) and 45% methanol fraction (**B**) (λ = 280 nm). Peak 1′: 4’-glucosylvitexin; Peak 2′: unknown compound; Peak 3′: rhamnosylvitexin; Peak 4′: unknown compound.

Table 2 showed tri-glycosides were the major components of the 60% methanol fraction, however, four contaminants, including 4’-glucosylvitexin and rhamnosylvitexin assigned to Peaks 1′ and 3′, as well as two unknown compounds assigned to Peaks 2′ and 4′, were observed in Figure 2A. For method B, the majority of flavonol glycosides were enriched in the 45% methanol fraction, with the concentration of TFG being 103.26 ± 4.31 µg/mL, and the concentrations of EGC and EC being lower than those of method A.

Table 2 showed the 45% methanol fraction of method B contained 3.06 ± 0.09 µg/mL of quercetin-3-*O*-glucosyl-rhamnosyl-galactoside, 16.47 ± 0.28 µg/mL of quercetin-3-*O*-glucosyl-rhamnosyl-glucoside (Q-glu-rha-glu), 4.39 ± 0.14 µg/mL of quercetin-3-*O*-rhamnosyl-rhamnosyl-galactoside (Q-gal-rha-rha), 10.86 ± 0.22 µg/mL of quercetin-3-*O*-rhamnosyl-rhamnosyl-glucoside (Q-glu-rha-rha), 2.14 ± 0.11 µg/mL of K-gal-rha-glu, 5.54 ± 0.17 µg/mL of kaempferol-*O*-rhamnosyl-rhamnosyl-galactoside (K-gal-rha-rha), 28.56 ± 0.76 µg/mL of K-glu-rha-glu and 28.85 ± 2.42 µg/mL of kaempferol-*O*-rhamnosyl-rhamnosyl-glucoside (K-glu-rha-rha), while the four contaminants (4’-glucosylvitexin, rhamnosylvitexin and two unknown compounds) were greatly reduced (Figure 2B). This was possibly due to the pre-cleaning effects of the 15% methanol and 30% methanol elutions, despite a cost of a small proportion of tri-glycosides. Polyamide has been used as a pretreatment column for production of tea extract by removing sugars, minerals, gallic acids or caffeine [19,20]. In the present study, the polyamide column was used for the enrichment of the flavonol glycoside fraction and preliminary purification in addition to removal of sugars, minerals, caffeine and gallic acids.

### 2.3. Impact of Flow Rate on the Composition of Flavonol Glycoside-Enriched Fraction

Table 3 shows the chemical compositions of 45% methanol fractions eluted at the flow rates of 2.5 mL/min, 5 mL/min and 8 mL/min. At the flow rates of 2.5 mL/min, the 45% methanol fraction contained 121.69 ± 4.99 µg/mL TFG, with the percentage of tri-glycosides being 96.9%. As the flow rate increased to 5 mL/min, the concentration of TFG significantly declined to 103.26 ± 4.31 µg/mL, corresponding to the percentage of tri-glycosides at 93.8%. There was no significant difference of TFG concentration observed in the 45% methanol fraction between 5 mL/min and 8 mL/min. In order to achieve a relatively high concentration of TFG for the subsequent preparative HPLC fractioning, 2.5 mL/min is the recommended flow rate.

### 2.4. Isolation and Characterization of Tri-Glycosides

**Figure 3:**
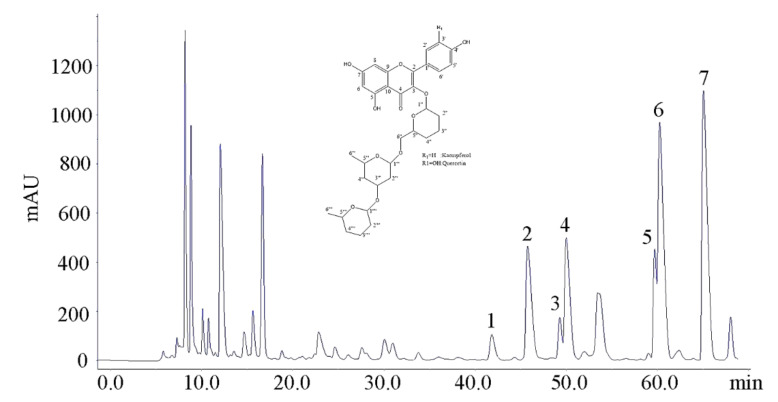
The preparative HPLC chromatogram of 45% methanol fraction. Peaks 1: Q-gal-rha-glu; Peak 2: Q-glu-rha-glu; Peak 3: Q-gal-rha-rha; Peak 4: Q-glu-rha-rha; Peak 5: K-gal-rha-rha; Peak 6: K-glu-rha-glu; Peak 7: K-glu-rha-rha based on the results of UPLC–DAD–MS/MS analysis. Inset: basic carbon skeleton of isolated tri-glycosides.

Figure 3 is the preparative HPLC chromatogram of the 45% methanol fraction isolated by Method B. According to the UPLC retention time of flavonol glycosides, the peaks from 40 min to 70 min were speculated as flavonol glycosides, which were termed Peaks 1, 2, 3, 4, 5, 6, 7 (Figure 3).

**Figure 4:**
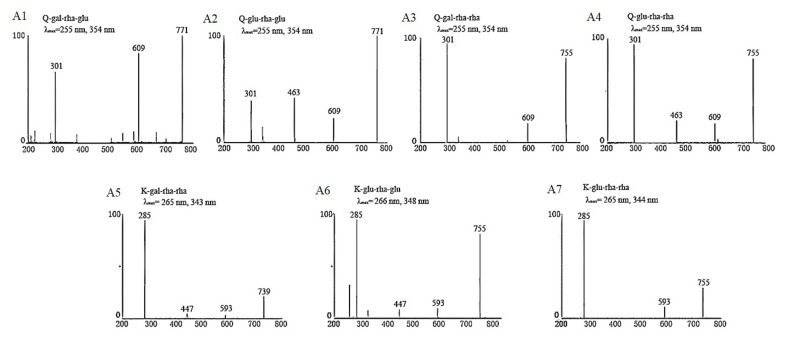
The basic characteristic information of isolated tri-glycosides.

In our previous studies [13,14], we established UPLC–DAD–MS/MS analysis methods for identification and quantification of flavonol glycosides in tea leaves based on the reported MS and MS/MS fragmentation data as well as UV λ_max_ of flavonol glycosides [18,19,20,21]. The developed method of reference [13] was used for determining the respectively collected fractions assigned to Peaks 1–7, which were identified as Q-gal-rha-glu, Q-glu-rha-glu, Q-gal-rha-rha, Q-glu-rha-rha, K-gal-rha-rha, K-glu-rha-glu and K-glu-rha-rha in order. The MS/MS spectra and UV λ_max_ of each isolated fraction were shown in Figure 4. The UV absorption spectra of these seven flavonol triglycosides had similar UV λ_max_ at 255~266 nm and 343~354 nm. The absorption peak around 255~266 nm was mainly attributed to the presence of benzene ring in the molecular structure of flavonol triglycosides, which was also impacted by the substituent groups through bathochromic/hypsochromic shift. The absorption peak around 343~354 nm was due to the presence of carbonyl group in the heterocyclic ring of flavonol glycoside, which is also the spectral characteristic distinguished from flavanols (mainly catechins). For MS/MS spectra, the fragments of 301 *m*/*z* and 285 *m*/*z* were assigned to the [M – H]^−^ of quercetin and kaempferol, respectively, and the decrements of 162 *m*/*z*, 146 *m*/*z* and 308 *m*/*z* between fragments were attributed to the losses of the moieties of hexose, rhamnose and the disaccharide of these two monosaccharides. The MS and MS/MS fragmentation data, as well as the UV λ_max_ of isolated compounds, verified the basic molecular structures of flavonol triglycosides.

In order to further validate the molecular and structural characterization, the fractions corresponding to the big Peaks 2, 4, 6, 7 were collected and freeze dried for ^1^H-NMR and ^13^C-NMR measurements. The ^1^H- and ^13^C-NMR data were shown as follows:

Peak 2: Q-glu-rha-glu: ^1^H-NMR (500 MHz, Methanol-d4) δ 7.69 [d, *J* = 2.2 Hz, 1H, C(2′)-H], 7.63 [dd, *J* = 8.4, 2.2 Hz, 1H, C(6′)-H], 6.88 [d, *J* = 8.4 Hz, 1H, C(5′)-H], 6.42 [d, *J* = 2.1 Hz, 1H, C(8)-H], 6.22 [d, *J* = 2.1 Hz, 1H, C(6)-H], 5.09 [d, *J* = 7.7 Hz, 1H, C(1′′)-H], 4.56 [d, *J* = 1.8 Hz, 1H, C(1′′′)-H], 4.43 [d, *J* = 7.8 Hz, 1H, C(1′′′′)-H], 3.94 [dd, *J* = 3.4, 1.7 Hz, 1H, C(2′′′)-H], 3.81 [dd, 1H, C(6′′A)-H], 3.76 [dd, 1H, C(6′′′′A)-H], 3.73 [dd, 1H, C(6′′′′B)-H)], 3.64 [dd, 1H, C(3′′′)-H], 3.52 [dd, 1H, C(5′′′)-H], 3.48 [m, 1H, C(6′′)-H], 3.47 [m, 2H, C(2′′)-H, C(4′′′)-H], 3.44 [m, 1H, C(6′′)-H], 3.43 [m, 1H, C(3′′′′)-H], 3.41 [m, 1H, C(4′′′′)-H], 3.38 [m, 1H, C(4′′)-H], 3.34 [m, 1H, C(5′′)-H], 3.28 [m, 1H, C(2′′′′)-H], 3.25 [m, 1H, C(5′′′′)-H], 1.11 [d, *J* = 6.0 Hz, 3H, C(6′′′)-H]. ^13^C NMR (126 MHz, Methanol-*d*_4_) δ 179.39 [C-4], 166.06 [C-7], 162.97 [C-5], 159.38 [C-2], 158.57 [C-9], 149.78 [C-4′], 145.81 [C-3′], 135.70 [C-3], 123.52 [C-6′], 123.10 [C-1′], 117.79 [C-2′], 116.06[C-5′], 105.62 [C-1′′′′,C-1′′], 104.91 [C-10], 102.35 [C-1′′′], 99.98 [C-6], 94.97 [C-8], 83.09 [C-3′′′], 78.20 [C-5′′], 77.58 [C-5′′′′, C-3′′′′], 77.19 [C-3′′], 75.72 [C-2′′], 75.46 [C-2′′′′], 72.59 [C-4′′′, C-4′′], 71.37 [C-2′′′], 70.85 [C-4′′′′], 69.38 [C-5′′′], 68.81 [C-6′′], 62.05 [C-6′′′′], 17.94 [C-6′′′].

Peak 4: Q-glu-rha-rha: ^1^H-NMR (500 MHz, Methanol-*d*_4_) δ 7.69 [d, *J* = 2.2 Hz, 1H,C(2′)-H], 7.64 [dd, *J* = 8.4, 2.2 Hz, 1H, C(6′)-H], 6.89 [d, *J* = 8.4 Hz, 1H, C(5′)-H], 6.40 [d, *J* = 2.1 Hz, 1H, C(8)-H], 6.22 [d, *J* = 2.1 Hz, 1H, C(6)-H], 5.09 [d, *J* = 7.7 Hz, 1H, C(1′’)-H], 4.98 [d, *J* = 1.7 Hz, 1H, C(1′′′′)-H], 4.51 [d, *J* = 1.7 Hz, 1H, C(1′′′)-H], 3.95 [dd, 1H, C(2′′′)-H], 3.81 [dd, 1H,C-(6′′a)], 3.74 [dd, 1H,C(5′′′′)-H], 3.72 [q, 1H, C-(2′′′′)-H], 3.68 [m, 1H,C(3′′′′)-H], 3.63 [dd, 1H, C(3′′′)-H], 3.52 [dd, 1H, C(5′′′)-H], 3.47 [t, 1H, C(4′′′)-H], 3.43 [m, 1H, C(2′′)-H], 3.41 [m, 1H, C(3′′)-H], 3.37 [m, 1H, C(4′′)-H], 3.34 [m, 1H, C(5′′)-H], 3.34 [d, 1H, C(5′′)-H], 3.27 [t, 1H, C(4′′′)-H], 1.17 [d, *J* = 6.2 Hz, 3H, C(6′′′)-H], 1.13 [d, *J* = 6.1 Hz, 3H, C(6′′′′)-H]. ^13^C NMR (126 MHz, Methanol-*d*_4_) δ 179.43 [C-4], 166.28 [C-7], 163.01 [C-5], 159.38 [C-2], 158.58 [C-9], 149.84 [C-4′], 145.81 [C-3′], 135.78 [C-3], 123.56 [C-6′], 123.12 [C-1′], 117.79 [C-2′], 116.10 [C-5′], 105.62 [C-10], 105.02 [C-1′′], 103.87 [C-1′′′′], 102.46 [C-1′’’], 100.09 [C-6], 95.01 [C-8], 79.56 [C-3′′′], 78.24 [C-5′′], 77.21 [C-3′′], 75.77 [C-2′′], 74.13 [C-4′′′′], 73.26 [C-2′′′′], 72.19 [C-4′′′′, C-4′′], 71.94 [C-3′′′′], 71.42 [C-2′′′], 69.99 [C-5′′′′, C-5′′′], 68.73 [C-6′′], 17.93 [C-6′′′′, C-6′′′].

Peak 6: K-glu-rha-glu: ^1^H-NMR (500 MHz, Methanol-*d*_4_) δ 8.07 [d, 2H, C(2′)-H, C(6′)-H], 6.90 [d, 2H, C(3′)-H, C(5′)-H], 6.42 [d, *J* = 2.1 Hz, 1H, C(8)-H], 6.22 [d, *J* = 2.1 Hz, 1H, C(6)-H], 5.11 [d, *J* = 7.3 Hz, 1H, C(1′′)-H], 4.55 [d, *J* = 1.7 Hz, C(1′′′)-H], 4.39 [d, *J* = 7.7 Hz, 1H, C(1′′′′)-H], 3.93 [dd, 1H, C(2′′′)-H], 3.81 [dd, 1H, C(6′′A)-H], 3.73 [dd, 1H, C(6′′′′B)-H)], 3.64 [dd, 1H, C(3′′′)-H], 3.57 [dd, 1H, C(3′′′)-H], 3.51 [m, 1H, C(5′′′)-H], 3.48 [m, 1H, C(6′′)-H], 3.47 [m, 2H, C(2′′)-H, C(4′′′)-H], 3.44 [m, 1H, C(6′′)-H], 3.43 [m, 1H, C(3′′′′)-H], 3.41 [m, 1H, C(4′′′′)-H], 3.38 [m, 1H, C(4′′)-H], 3.34 [m, 1H, C(5′′)-H], 3.28 [m, 1H, C(2′′′′)-H], 3.25 [m, 1H, C(5′′′′)-H], 1.11 [d, *J* = 6.0 Hz, 3H, C(6′′′)-H]. ^13^C NMR (126 MHz, Methanol-*d*_4_) δ 179.42 [C-4], 166.18 [C-7], 163.01 [C-5], 161.50 [C-4′], 159.48 [C-2], 158.64 [C-9], 135.59 [C-3], 132.46 [C-2′, C-6′], 122.78 [C-1′], 116.17 [C-3′, C-5′], 105.67 [C-1′′, C-1′′′′], 104.72 [C-10], 102.33 [C-1′′′], 100.06 [C-6], 95.06 [C-8], 83.19 [C-3′′′], 78.19 [C-5′′], 77.58 [C-5′′′′, C-3′′′′], 77.15 [C-3′′], 75.77 [C-2′′], 75.46 [C-2′′′′], 72.57 [C-4′′′], 71.54 [C-4′′], 71.30 [C-2′′′], 70.85 [C-4′′′′], 69.43 [C-5′′′], 68.95 [C-6′′], 62.07 [C-6′′′′], 17.97 [C-6′′′].

Peak 7: K-glu-rha-rha: ^1^H-NMR (500 MHz, Methanol-*d*_4_) δ 8.07 [d, 2H, C(2′)-H, C(6′)-H], 6.90 [d, 2H, C(3′)-H, C(5′)-H], 6.40 [d, *J* = 2.0 Hz, 1H, C(8)-H], 6.22 [d, *J* = 2.1 Hz, 1H, C(6)-H], 5.11 [d, *J* = 7.3 Hz, 1H, C(1′′)-H], 4.98 [d, *J* = 1.7 Hz, 1H, C(1′′′′)-H], 4.51 [d, *J* = 1.7 Hz, 1H, C(1′′′)-H], 3.94 [dd, 1H, C(2′′′)-H], 3.81 [dd, 1H, C-(6′′a)], 3.74 [dd, 1H, C(5′′′′)-H], 3.72 [q, 1H, C-(2′′′′)-H], 3.68 [m, 1H, C(3′′′′)-H], 3.63 [dd, 1H, C(3′′′)-H], 3.52 [dd, 1H, C(5′′′)-H], 3.47 [t, 1H, C(4′′′)-H], 3.43 [m, 1H, C(2′′)-H], 3.41 [m, 1H, C(3′′)-H], 3.37 [m, 1H, C(4′′)-H], 3.34 [m, 1H, C(5′′)-H], 3.27 [t, 1H, C(4′′′)-H], 1.17 [d, *J* = 6.3 Hz, 3H, C(6′′′)-H], 1.13 [d, *J* = 6.1 Hz, 3H, C(6′′′′)-H]. ^13^C NMR (126 MHz, Methanol-*d*_4_) δ 179.40 [C-4], 166.13 [C-7], 162.99 [C-5], 161.48 [C-4′], 159.48 [C-2], 158.58 [C-9], 135.62 [C-3], 132.41 [C-2′, C-6′], 122.77 [C-1′], 116.15 [C-3′, C-5′], 105.66 [C-10], 104.77 [C-1′′], 103.87 [C-1′′′′], 102.43 [C-1′′′], 100.07 [C-6], 95.05 [C-8], 79.40 [C-3′′′], 78.16 [C-5′′], 77.20 [C-3′′], 75.78 [C-2′′], 74.10 [C-4′′′′], 73.22 [C-2′′′′], 72.18 [C-4′′′′, C-4′′], 71.92 [C-3′′′′], 71.51 [C-2′′′], 70.01 [C-5′′′′, C-5′′′], 68.81 [C-6′′], 17.94 [C-6′′′′, C-6′′′].

The inset of Figure 3 showed the basic carbon skeleton of isolated tri-glycosides. The ^1^H- and ^13^C-NMR data of Q-glu-rha-glu, Q-glu-rha-rha, K-glu-rha-glu and K-glu-rha-rha were consistent with the reported data [9,17,20]. For ^1^H-NMR, the chemical shifts of protons on benzene ring were above 6 ppm. The chemical shifts of protons within the range of 6–4 ppm were assigned to the protons at the glycosidic linkages of flavonol triglycosides [C(1′′)-H, C(1′′′)-H, C(1′′′′)-H], which were higher than the chemical shift values of protons on sugar moieties. For instance, 5.09 ppm (d, *J* = 7.7 Hz), 4.98 ppm (d, *J* = 1.7 Hz) and 4.51 ppm (d, *J* = 1.7 Hz) were corresponding to the protons at the glycosidic linkages of Q-glu-rha-rha, which were higher than those of protons on sugar moieties ranging from 3.95 ppm to 1.13 ppm. The relatively higher chemical shift values of anomeric protons are attributed to the deshielding effect. The ^13^C-NMR spectra of four isolated flavonol triglycosides were in general agreement with the references [9,17,20]. There were 0.10 mg Q-gal-rha-glu, 0.85 mg Q-glu-rha-glu, 0.13 mg Q-gal-rha-rha, 0.46 mg Q-glu-rha-rha, 0.25 mg K-gal-rha-rha, 1.53 mg K-glu-rha-glu and 1.56 mg K-glu-rha-rha isolated from 1 g of dry tea, with the purities of isolated flavonol triglycosides being above 95% based on HPLC. The production yield of TFG was 0.487% in total, which was much higher than the reported production yield [16].

## 3. Materials and Methods

### 3.1. Chemicals

Green tea (dry leaves) was purchased from the local tea market (Hubei Province, China), the chemical composition of which was shown in Table 4. Standard compounds: (−)-gallocatechin (≥98%, GC), (−)-epigallocatechin (≥98%, EGC), (+)-catechin (≥98%, C), EC (≥97%), EGCG (≥98%), (−)-gallocatechin gallate (≥98%, GCG), ECG (≥98%), (−)-catechin gallate (≥98%, CG), theobromine (≥95%), theophylline (≥95%), caffeine (≥95%) were purchased from Sigma (Sigma-Aldrich, St. Louis, MO, USA). Myricetin-3-*O*-glucoside (≥99%, M-glu), quercetin-3-*O*-glucoside (≥99%, Q-glu), kaempferol-3-*O*-glucoside (≥98%, K-glu) were purchased from Chengdu DeSiTe Biological Technology Co., Ltd. (Chengdu, China). HPLC grade methanol and acetonitrile were purchased from Sigma-Aldrich (Shanghai, China). Polyamide (particle size 74–174 µm) and formic acid (≥99%, HPLC grade) were bought from Aladdin (Shanghai, China). The Ultrapure water was prepared by an EASYPure II UV Ultra Pure Water System (Barnstead International, Dubuque, IA, USA).

### 3.2. Preparation of Tea Extract

Green tea was ground into powder and sieved through 100 µm. Five grams of green tea powder was extracted with 40 mL boiling water at 100 °C and 100 rpm for 30 min. After centrifugation at 2292× *g* and 4 °C for 5 min, the supernatant was collected and made up to 30 mL with water.

### 3.3. Preparation of Flavonol Glycoside-Enriched Fraction Using Polyamide Column

#### 3.3.1. Polyamide Column Packing

Polyamide was soaked in methanol for 24 h, and then was packed in a column (1 cm × 6.4 cm). The column was pre-equilibrated with 2% NaOH solution, 2% HCl solution and water prior to use.

#### 3.3.2. Effect of Uploading Volume

In order to achieve the maximum uploading volume of tea extract, different volumes of tea extract (1 mL, 2.5 mL and 5 mL) were, respectively, uploaded onto polyamide column. The effluent was collected and submitted to UPLC–DAD–MS/MS analysis.

#### 3.3.3. Effect of Different Elution Methods

Tea extract (2.5 mL) was uploaded onto the polyamide column. Two different elution methods were used for preparing flavonol glycoside-enriched fraction: Method A: 20 mL of water → 20 mL of 60% methanol solution (*v*/*v*) → 20 mL of methanol; Method B: 20 mL of water → 20 mL of 15% methanol solution (*v*/*v*) → 20 mL of 30% methanol solution (*v*/*v*) → 20 mL of 45% methanol solution (*v*/*v*) → 20 mL of 70% methanol solution (*v*/*v*) → 20 mL of methanol. Flow rate was 5 mL/min. Each fraction was collected for chemical analyses.

#### 3.3.4. Effect of Flow Rate

Tea extract (2.5 mL) was uploaded onto the polyamide column and subsequently eluted by Method B at different flow rates of 2.5 mL/min, 5 mL/min and 8.00 mL/min. Each fraction was collected for chemical analyses.

### 3.4. Preparative HPLC

The flavonol glycoside-enriched fraction (uploading volume of 2.5 mL, 45% methanol fraction by Method B) was collected and concentrated by a rotary evaporator. The concentrate was reconstituted with 4 mL water and then filtered through a 0.22 µm membrane before injection into the preparative HPLC AKTA purifier UPC100 (GE Healthcare Bio-Sciences AB, Sweden). The preparative HPLC conditions were: YMC-Pack ODS-A column (5 µm, 250 × 10 mm), injection volume 0.5 mL, mobile phase A: acetonitrile/acetic acid/water = 3.0 : 0.5 : 96.5 (*v*/*v*/*v*), mobile phase B: water/acetonitrile/acetic acid = 30.0 : 0.5 : 69.5 (*v*/*v*/*v*), gradient elution: maintaining 55% A and 45% B during the first 35 min, and then linearly increasing to 35% A and 65% B till 55 min, and increasing to 100% B till 70 min, flow rate 2 mL/min. The fractions corresponding to Peaks 1–7 were collected for chemical analyses.

### 3.5. UPLC–DAD–MS/MS Analysis of Flavonol Glycosides

After centrifugation at 13,201× *g* and 4 °C for 20 min, the supernatants were submitted to UPLC–DAD–MS/MS (Waters Corporation, Milford, MA, USA) according to the reported method [13]. Briefly, the UPLC conditions were: CORTECS T3 column (2.1 mm × 100 mm, 1.6 μm), column temperature 25 °C, injection volume 2 μL, mobile phase A = 0.1% formic acid solution, mobile phase B = acetonitrile. The gradient elution program and detection method were exactly according to the method [13]. An electrospray ionization (ESI) technique in a negative ion mode was used for MS scan, with the scan scale from 200 *m*/*z* to 1000 *m*/*z*. The MS conditions were set as follows: cone voltage 30 V, extractor voltage 3.0 V, capillary voltage 3 kV, ion source temperature 150 °C, desolvation temperature 350 °C, desolvation gas flow 400 L/h. External standards including M-glu, Q-glu, and K-glu were respectively used for quantifying myricetin glycosides, quercetin glycosides and kaempferol glycosides based on UPLC chromatogram.

### 3.6. HPLC Analysis

The concentrations of catechin compounds and alkaloids were analyzed by LC 20A HPLC (Shimadzu Co., Kyoto, Japan) according to our previously published method [22]. Briefly, the HPLC conditions were as follows: injection volume 10 µL, Zorbax 5 µm TC-C_18_ (2) column (250 mm × 4.6 mm, Agilent Technologies Inc., CA, USA), column temperature 28 °C, mobile phase A: acetonitrile/acetic acid/water = 3 : 0.5 : 96.5 (*v*/*v*/*v*), mobile phase B: acetonitrile/acetic acid/water = 30 : 0.5 : 69.5 (*v*/*v*/*v*), gradient elution: linearly increasing from 30% B to 85% B during the first 35 min, and then holding at 85% B for another 5 min, detection wavelength 280 nm.

### 3.7. ^1^H and ^13^C Nuclear Magnetic Resonance (NMR) Measurement

The fractions corresponding to Peaks 2, 4, 6, 7 on preparative HPLC were freeze dried for NMR measurement. Ten mg of the obtained freeze-dried compounds was suspended in 0.6 mL CD_3_OD for ^1^H- and ^13^C-NMR measurement. The spectra were recorded on a Bruker AVANCE III spectrometer at 500 MHz with a 5 mm DCH cryoprobe (Bruker, Wissembourg, France). Integration of the spectra was performed on Mestnova 9.0.1 (Mestrelab Research S. L., Santiago de Compostela, Spain).

### 3.8. Data Analysis

All analyses were carried out in triplicate. The results of above tests were expressed as the mean value ± SD. Statistical significance was carried out on SAS 9.4 software (SAS Institute Inc., Cary, NC, USA), using Tukey test. The structures of compounds were drawn by ChemOffice 2010 v12 (CambridgeSoft, USA).

## 4. Conclusions

The present study developed a two stepwise method for simultaneously preparing abundant flavonol triglycosides from tea leaves, comprising enrichment of flavonol glycosides by polyamide column and preparative HPLC isolation. Seven flavonol glycoside individuals were isolated and characterized based on MS, UV absorption and NMR spectra, namely, Q-gal-rha-glu, Q-glu-rha-glu, Q-gal-rha-rha, Q-glu-rha-rha, K-gal-rha-rha, K-glu-rha-glu and K-glu-rha-rha. The production yield of TFG was 0.487% in total.

## Figures and Tables

**Table 1:** The effect of elution method on the chemical composition of different fractions (µg/mL).

Fractions	TB	EGC	Caffeine	EC	EGCG	GCG	ECG	TFG
Method A								
Aqueous	18.73 ± 0.54	ND	254.52 ± 6.37	ND	ND	ND	ND	ND
60% Methanol	ND	240.79 ± 12.19	ND	35.11 ± 2.39	ND	ND	ND	140.13 ± 1.56
100% Methanol	ND	287.64 ± 10.49	ND	24.35 ± 0.67	370.61 ± 7.89	ND	162.78 ± 3.10	48.11 ± 2.03
Method B								
Aqueous	19.27 ± 0.09	ND	261.08 ± 0.79	ND	ND	ND	ND	ND
15% Methanol	ND	ND	3.74 ± 0.20	ND	ND	ND	ND	ND
30% Methanol	ND	ND	ND	ND	ND	ND	ND	17.64 ± 2.02
45% Methanol	ND	211.88 ± 11.73	ND	24.20 ± 0.75	ND	ND	ND	103.26 ± 4.31
70% Methanol	ND	320.36 ± 5.33	ND	61.64 ± 0.97	18.93 ± 2.59	ND	ND	61.46 ± 2.57
100% Methanol	ND	ND	ND	ND	784.26 ± 24.72	21.16 ± 1.29	246.79 ± 6.26	9.17 ± 1.56

TB: theobromine. EGC: (−)-epigallocatechin. EC: (−)-epicatechin. EGCG: (−)-epigallocatechin gallate. GCG: (−)-Gallocatechin gallate. ECG: (−)-epicatechin gallate. TFG: total flavonol glycosides. ND: not detected.

**Table 2:** The effect of elution method on the composition of flavonol glycosides (µg/mL).

	Method A		Method B			
	60% Methanol	Methanol	30% Methanol	45% Methanol	70% Methanol	Methanol
M-gal	ND	9.42 ± 0.12	ND	ND	4.06 ± 0.73	5.11 ± 0.49
M-glu	ND	7.31 ± 0.02	ND	ND	5.23 ± 0.18	2.06 ± 0.27
Q-glu-rha-gal	4.82 ± 0.06	0.71 ± 0.50	ND	3.06 ± 0.09	2.29 ± 0.11	ND
Q-glu-rha-glu	20.76 ± 0.04	1.16 ± 0.06	2.76 ± 0.39	16.47 ± 0.28	3.4 ± 0.24	ND
Q-gal-rha-rha	7.86 ± 0.05	2.44 ± 0.10	ND	4.39 ± 0.14	6.43 ± 0.24	0.15 ± 0.21
Q-glu-rha-rha	13.27 ± 0.02	0.84 ± 0.04	0.88 ± 0.16	10.86 ± 0.22	2.87 ± 0.12	ND
Q-glu-rha	3.60 ± 0.10	0.91 ± 0.04	ND	2.04 ± 0.06	2.21 ± 0.15	ND
K-gal-rha-glu	3.42 ± 0.07	ND	0.84 ± 0.08	2.14 ± 0.11	0.38 ± 0.05	ND
Q-gal	ND	2.74 ± 0.07	ND	ND	1.77 ± 0.07	0.63 ± 0.46
Q-glu	ND	6.93 ± 0.04	ND	ND	6.13 ± 0.02	1.22 ± 0.13
K-gal-rha-rha	7.30 ± 0.02	0.25 ± 0.18	1.18 ± 0.12	5.54 ± 0.17	1.05 ± 0.08	ND
K-glu-rha-glu	37.76 ± 0.13	1.61 ± 0.28	7.41 ± 0.76	28.56 ± 0.76	4.59 ± 0.24	ND
K-glu-rha-rha	38.00 ± 0.15	2.11 ± 0.10	4.56 ± 0.51	28.85 ± 2.42	6.41 ± 0.10	ND
K-gal	ND	1.10 ± 0.04	ND	ND	1.38 ± 0.08	ND
K-glu-rha	2.09 ± 0.03	0.14 ± 0.20	ND	1.35 ± 0.07	1.31 ± 0.03	ND
K-glu	1.25 ± 0.88	10.46 ± 0.23	ND	ND	11.96 ± 0.16	ND

ND: not detected.

**Table 3:** The effect of flow rate on the chemical compositions of 45% methanol fractions (µg/mL).

	Q-gal-rha-glu	Q-glu-rha-glu	Q-gal-rha-rha	Q-glu-rha-rha	Q-glu-rha	K-gal-rha-glu	K-gal-rha-rha	K-glu-rha-glu	K-glu-rha-rha	K-glu-rha	TFG
2.5 mL/min	3.56 ± 0.11 ^a^	19.27 ± 0.77 ^a^	4.38 ± 0.17 ^a^	12.46 ± 0.38 ^a^	2.19 ± 0.18 ^a^	2.42 ± 0.12 ^a^	6.62 ± 0.28 ^a^	33.84 ± 1.62 ^a^	35.38 ± 1.32 ^a^	1.57 ± 0.04 ^a^	121.69 ± 4.99 ^a^
5 mL/min	3.06 ± 0.09 ^b^	16.47 ± 0.28 ^b^	4.39 ± 0.14 ^a^	10.86 ± 0.22 ^b^	2.04 ± 0.06 ^a^	2.14 ± 0.11 ^b^	5.54 ± 0.17 ^b^	28.56 ± 0.76 ^b^	28.85 ± 2.42 ^b^	1.35 ± 0.07 ^b^	103.26 ± 4.31 ^b^
8 mL/min	3.08 ± 0.19 ^b^	15.66 ± 0.2 ^c^	4.25 ± 0.53 ^a^	10.12 ± 0.35 ^b^	2.15 ± 0.37 ^a^	2.38 ± 0.28 ^ab^	5.38 ± 0.06 ^b^	27.86 ± 0.41 ^b^	28.9 ± 0.58 ^b^	1.34 ± 0.15 ^b^	101.11 ± 3.12 ^b^

TFG: total flavonol glycosides. Data with different alphabetic letters in a same column were significantly different at *p* = 0.05.

**Table 4:** The chemical composition of tea leaves (mg/g).

Compounds	Abbreviation	Content
Theobromine		1.19 ± 0.03
Theophylline		0.05 ± 0.01
Caffeine		18.09 ± 0.50
(−)-gallocatechin	GC	2.97 ± 0.08
(−)-Epigallocatechin	EGC	26.62 ± 1.29
(+)-catechin	C	1.49 ± 0.04
(−)-Epicatechin	EC	5.01 ± 0.14
(−)-Epigallocatechin gallate	EGCG	74.94 ± 2.07
(−)-Gallocatechin gallate	GCG	3.75 ± 0.10
(−)-Epicatechin gallate	ECG	19.64 ± 0.54
(+)-catechin gallate	CG	0.48 ± 0.01
Myricetin-3-*O*-rhamnosyl-glucoside	M-glu-rha	0.11 ± 0.01
Myricetin-3-*O*-galactoside	M-gal	0.44 ± 0.05
Myricetin-3-*O*-glucoside	M-glu	0.36 ± 0.04
Quercetin-3-*O*-glucosyl-rhamnosyl-galactoside	Q-gal-rha-glu	0.32 ± 0.01
Quercetin-3-*O*-glucosyl-rhamnosyl-glucoside	Q-glu-rha-glu	1.15 ± 0.04
Quercetin-3-*O*-rhamnosyl-rhamnosyl-galactoside	Q-gal-rha-rha	0.54 ± 0.02
Quercetin-3-*O*-rhamnosyl-rhamnosyl-glucoside	Q-glu-rha-rha	0.73 ± 0.02
Quercetin-3-*O*-rhamnosyl-glucoside	Q-glu-rha	0.28 ± 0.01
Kaempferol-3-*O*-glucosyl-rhamnosyl-galactoside	K-gal-rha-glu	0.16 ± 0.01
Quercetin-3-*O*-galactoside	Q-gal	0.13 ± 0.02
Quercetin-3-*O*-glucoside	Q-glu	0.33 ± 0.04
Kaempferol-*O*-rhamnosyl-rhamnosyl-galactoside	K-gal-rha-rha	0.41 ± 0.01
Kaempferol-3-*O*-glucosyl-rhamnosyl-glucoside	K-glu-rha-glu	2.06 ± 0.05
Kaempferol-*O*-rhamnosyl-rhamnosyl-glucoside	K-glu-rha-rha	2.13 ± 0.02
Kaempferol-3-*O*-galactoside	K-gal	0.08 ± 0.01
Kaempferol-3-*O*-rhamnosyl-glucoside	K-glu-rha	0.14 ± 0.01
Kaempferol-3-*O*-glucoside	K-glu	0.57 ± 0.08
